# Oncogenic STAT5 signaling promotes oxidative stress in chronic myeloid leukemia cells by repressing antioxidant defenses

**DOI:** 10.18632/oncotarget.11480

**Published:** 2016-08-22

**Authors:** Jerome Bourgeais, Nicole Ishac, Magdalena Medrzycki, Marie Brachet-Botineau, Laura Desbourdes, Valerie Gouilleux-Gruart, Emmanuel Pecnard, Florence Rouleux-Bonnin, Emmanuel Gyan, Jorge Domenech, Frederic Mazurier, Richard Moriggl, Kevin D. Bunting, Olivier Herault, Fabrice Gouilleux

**Affiliations:** ^1^ CNRS UMR 7292, GICC, Université F Rabelais, Tours, France; ^2^ Aflac Cancer and Blood Disorders Center of Children's Healthcare of Atlanta and Emory University School of Medicine Atlanta, GA, USA; ^3^ CHRU de Tours, Laboratoire d’Immunologie, Tours, France; ^4^ CHRU de Tours, Service d’Hématologie Clinique et Thérapie Cellulaire, Tours, France; ^5^ CHRU de Tours, Service d’Hématologie Biologique, Tours, France; ^6^ University of Veterinary Medicine, Medical University of Vienna and Ludwig Boltzmann Institute for Cancer Research, Vienna, Austria

**Keywords:** STAT5, Bcr-Abl, chronic myeloid leukemia, oxidative stress, antioxidants

## Abstract

STAT5 transcription factors are frequently activated in hematopoietic neoplasms and are targets of various tyrosine kinase oncogenes. Evidences for a crosstalk between STAT5 and reactive oxygen species (ROS) metabolism have recently emerged but mechanisms involved in STAT5-mediated regulation of ROS still remain elusive. We demonstrate that sustained activation of STAT5 induced by Bcr-Abl in chronic myeloid leukemia (CML) cells promotes ROS production by repressing expression of two antioxidant enzymes, catalase and glutaredoxin-1(Glrx1). Downregulation of catalase and Glrx1 expression was also observed in primary cells from CML patients. Catalase was shown not only to reduce ROS levels but also, to induce quiescence in Bcr-Abl-positive leukemia cells. Furthermore, reduction of STAT5 phosphorylation and upregulation of catalase and Glrx1 were also evidenced in leukemia cells co-cultured with bone marrow stromal cells to mimic a leukemic niche. This caused downregulation of ROS levels and enhancement of leukemic cell quiescence. These data support a role of persistent STAT5 signaling in the regulation of ROS production in myeloid leukemias and highlight the repression of antioxidant defenses as an important regulatory mechanism.

## INTRODUCTION

The Signal Transducer and Activator of Transcription factors 5A and 5B are two closely related STAT family members that play a major role in normal and leukemic hematopoiesis [1]. Both proteins are crucial effectors of cytokine and/or growth factor-induced survival, proliferation and differentiation of hematopoietic cells. Tyrosine phosphorylation is a critical initial step in cytokine-dependent STAT activation and is regulated by JAK family members which bind to cytokine receptors. Phosphorylation of tyrosine residues Y694 (STAT5A) and Y699 (STAT5B) is a prerequisite for dimer formation and transcriptional activation of STAT5-regulated genes [2].

Persistent STAT5 phosphorylation is triggered by tyrosine kinase oncogenes (TKOs) such as Fms-like receptor tyrosine kinase 3 with internal tandem duplications (Flt3-ITD), Kit^D816V^, Bcr-Abl and JAK2^V617F^ which have been characterized in various myeloid malignancies [3–7]. STAT5 is a crucial downstream effector of Bcr-Abl and JAK2^V617F^, the major transforming agents in Philadelphia-positive (Ph+) CML and Philadelphia-negative (Ph-) myeloproliferative neoplasms respectively. Inhibition of STAT5 phosphorylation interrupts the transforming potential of these TKOs and the induction of leukemia in mouse models [8–11]. Previous reports indicated that STAT5 is a direct target of these TKOs, but additional events linked to cell transformation are probably involved in STAT5 activation [12, 13]. It was shown, for instance, that ROS, which are generated in oncogene-transformed cells regulate different redox-sensitive signaling pathways by inducing the reversible oxidation of tyrosine phosphatases, protein kinases and transcription factors [14, 15]. ROS-dependent inhibition of tyrosine phosphatases might contribute to enhanced tyrosine phosphorylation of signaling proteins including STATs [16, 17]. It is now well recognized that ROS are important initiators and promoters of carcinogenesis and contribute to tumor progression. High levels of ROS promote survival, proliferation, genomic instability and mutagenesis leading to disease progression and drug resistance [18–21]. Oxidative stress is due to an excessive cellular ROS production and/or a deficiency in antioxidant defenses. TKOs were shown to stimulate the production of intracellular ROS in leukemic cells [21–23]. ROS contribute to the regulation of signaling pathways in leukemic cells but elevated levels of ROS may also be dependent on deregulated pathways. Constitutive activation of the PI3-kinase/Akt pathway was shown to increase ROS levels in Bcr-Abl-transformed cells [24]. Bcr-Abl and Flt3-ITD also induce intracellular ROS production through STAT5 signaling [21, 25, 26]. However the mechanisms involved in STAT5-mediated induction of ROS remains unclear. In Flt3-ITD-expressing cells, STAT5 has been proposed to regulate activity of NADPH oxidase, one major cellular source of ROS, via distinct mechanisms [21, 26]. In sharp contrast, it was shown that STAT5A knockdown induced ROS production in Bcr-Abl^+^ hematopoietic stem/progenitor cells supporting a protective role of STAT5A against oxidative stress in these primary cells [27]. A similar protective effect was observed in pre-B leukemic cells [28]. These controversial data argue for different functions of STAT5 in the regulation of ROS levels, acting either as a repressor or as an inducer through mechanisms that remain unresolved. We show that oncogenic activation of STAT5 induced by Bcr-Abl in CML cells enhances ROS levels through the repression of catalase and glutaredoxin-1 (Glrx1), two enzymes involved in antioxidant defenses. These data suggest that deregulated STAT5 activity directly affects the balance between ROS generation and scavenging to promote oxidative stress in myeloid leukemias.

## RESULTS

### STAT5 promotes ROS production in Ph^+^ leukemia cells

**Figure 1:**
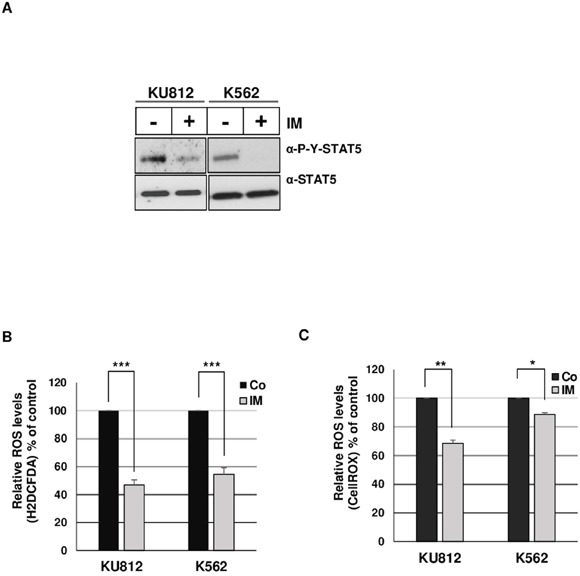
Bcr-Abl induces ROS production in leukemia cells **A**. Lysates from KU812 and K562 cells treated (+) or not (−) with 1 μM of IM for 15 h were analyzed by Western blotting with indicated antibodies. Results are the mean of 3 independent experiments. **B**. Statistical analysis showing relative ROS levels (% of control) detected in KU812 and K562 cells treated or not (Co) with 1 μM IM for 15 h. Cells were incubated with the ROS sensitive fluorescent dye H2DCFDA (5 μM) and intracellular ROS levels were monitored by flow cytometry (n=11 data are mean ± SEM,*** p <0.001). **C**. Statistical analysis showing relative ROS levels (% of control) detected in KU812 and K562 cells treated or not (Co) with 1 μM IM for 15 h and stained with the ROS sensitive probe CellROX (n=3, data are mean ± SEM. *p<0.05; **p<0.01).

**Figure 2:**
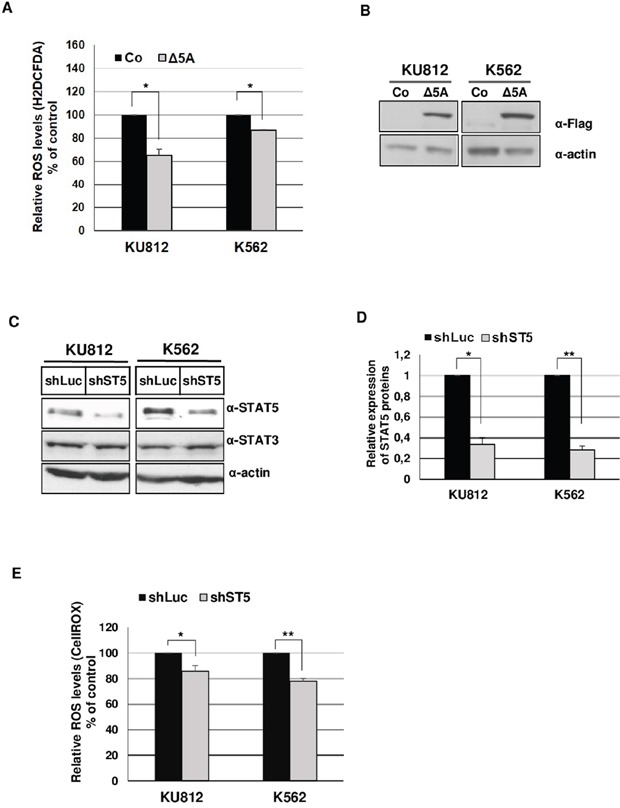
STAT5 promotes ROS production in Bcr-Abl+ leukemia cells **A**. KU812, K562 cell lines were transfected with a dominant negative Flag-STAT5AΔ749-ΔCD4 (Δ5A) bicistronic construct or empty vector (Co). Cells were next incubated with H2DCFDA (5μM) and APC-conjugated anti-CD4 antibody to determine ROS levels in CD4^+^ transfected cells. (n=3, data are mean ± SEM, * p <0.05). **B**. Extracts from transfected KU812 and K562 cells were prepared and analyzed by Western blotting with an anti-Flag antibody to verify expression of the dominant negative STAT5AΔ749 mutant (Δ5A). Actin served as a loading control (α-actin). **C**. KU812 and K562 cells were transfected with shST5/GFP or control shLuc/GFP vectors. Cell lysates were prepared 3 days after transfection and analyzed by Western blotting with indicated antibodies (n=3).**D**. Quantification of Western blot (ImageJ software) was performed to determine the relative expression of STAT5 (ratio STAT5/actin) in cells transfected with shST5 or shLuc expression vectors (n=3). **E**. KU812 and K562 cells transfected with shST5/GFP or control shLuc/GFP vectors were stained with CellROX at 3 days post-transfection to quantify ROS levels in GFP^+^ cells (n=5, data are mean ± SEM, *p<0.05; **p<0.01).

The main aim of this work was to elucidate the mechanisms involved in STAT5-dependent regulation of ROS production in Bcr-Abl^+^ leukemia cells. Initial experiments were carried out to demonstrate that inhibition of Bcr-Abl kinase activity in KU812 and K562 cell lines resulted in decreased ROS production following treatment with the Bcr-Abl kinase inhibitor: Imatinib Mesylate (IM) (Figure 1). KU812 and K562 cells treated with 1 μM IM, a concentration high enough to suppress STAT5 tyrosine phosphorylation (Figure 1A), had a lower level of intracellular ROS compared to non-treated cells as measured using the ROS-sensitive probes H2DCFDA (53% and 45% decrease in KU812 and K562 cells respectively) and CellROX (32% and 12% decrease) (Figure 1B-1C). To determine whether STAT5 activation is involved in ROS production, we examined the functional consequence of STAT5 inhibition on the regulation of ROS levels in Bcr-Abl^+^ cells. Because STAT5 inhibition could indirectly affect ROS levels through induction of apoptosis [29], we performed transient transfections assays to determine, shortly after transfection, the direct effect of STAT5 inhibition on ROS production. In a first set of experiments, KU812, and K562 cells were electroporated with a bicistronic vector allowing expression of a dominant negative Flag-STAT5AΔ749 mutant (Δ5A) and a truncated and inactive version of the cell surface expressing CD4 antigen (ΔCD4). Cells were next labeled with an APC-conjugated anti-CD4 antibody and the ROS sensitive probe H2DCFDA to determine ROS production in transfected ΔCD4^+^ cell populations. ROS levels were decreased in KU812 and K562 cells overexpressing STAT5Δ749 when compared to cells transfected with an empty vector (Figure 2A). Expression levels of STAT5AΔ749 were found similar in KU812 and K562 cells as determined by Western blot analysis (Figure 2B). We then compared the effects of STAT5 knockdown on the regulation of ROS levels in these different cell types. Cells were electroporated with vectors allowing expression of a STAT5 shRNA (shST5) targeting both STAT5 isoforms or a control shRNA (shluc) and the GFP protein. STAT5 but not STAT3 was specifically targeted by shST5 as determined by Western blot and knockdown efficiency was evaluated by band intensity quantification (Figure 2C and 2D). As the emission spectrum of GFP and H2DCFDA overlap, transfected cells were then stained with the CellROX probe to measure ROS levels in GFP^+^ cell populations (Figure 2E). Inhibition of STAT5 expression significantly decreased ROS production indicating that STAT5 activity directly enhanced ROS levels in Bcr-Abl-expressing cells.

### Persistent STAT5 activity inhibits expression of catalase and Glrx1 in CML cells

**Figure 3:**
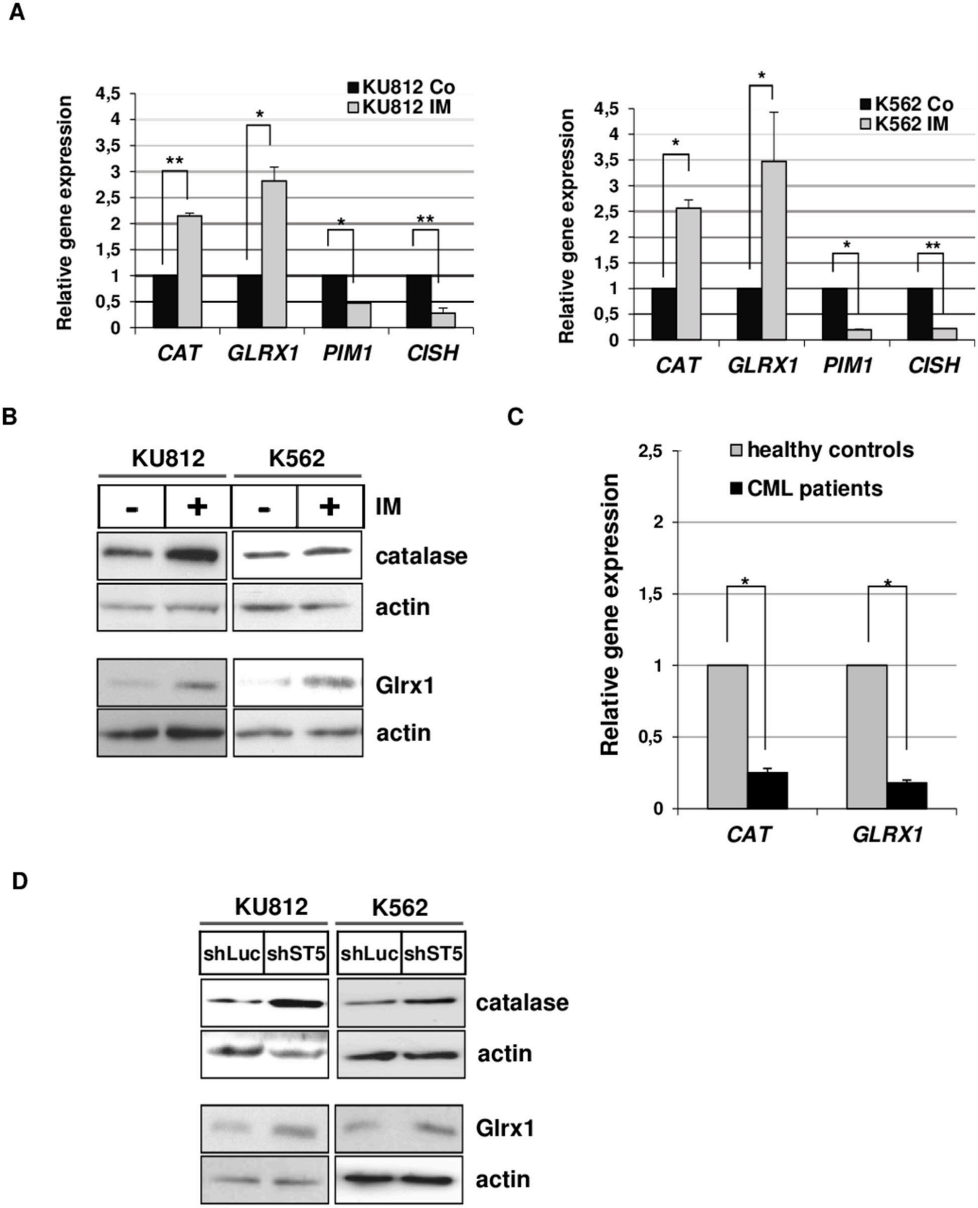
STAT5-dependent repression of Catalase and GLRX1 expression in CML cells **A**. qRT-PCR analysis of *CAT*, *GLRX1, CISH and PIM1* transcripts in KU812 (left) and K562 (right) cells treated or not with IM (1μM) for 15 h. Results are presented as the fold changes in gene expression in IM-treated cells normalized to internal control genes (*GAPDH* and *ACTB*) and relative to untreated cells (normalized to 1) (n=3 in triplicates, data are mean +/− SEM. **p<0.01; *p<0.05). **B**. Protein extracts from KU812 and K562 cells treated or not with IM (1μM) were analyzed by Western blotting to detect catalase and Glrx1 protein expression. Actin served as a loading control. (n=3). **C**. qRT-PCR analysis of *CAT* and *GLRX1* expression in leukemia cells from CML patients (n=35) and peripheral mononuclear (PMN) cells from healthy donors (n=10). **D**. Levels of catalase and Glrx1 proteins in KU812 and K562 cells transfected with shST5/GFP or shLuc/GFP vectors were also determined by Western blot analysis (n=3).

Oxidative stress is the result of an imbalance between ROS generation and antioxidant defenses [30]. We then tested whether STAT5 activity could regulate expression of detoxifying enzymes and scavengers in Bcr-Abl expressing cells by qRT-PCR experiments. We included the two STAT5-regulated genes: *PIM1* and *CISH* as positive controls in these assays (Supplementary Table S1). First, we characterized the antioxidant gene expression profile in Bcr-Abl^+^ cells treated or not with IM. Among the 28 main antioxidant genes tested, expression of two genes: *Catalase* (*CAT*) and *Glutaredoxin-1(GLRX1)* were significantly upregulated in KU812 and K562 cells (Supplementary Figure S1 and S2). We found that IM induced the expression of *CAT* (2.1x and 2.5x fold increase in KU812 and K562 cells, respectively) and *GLRX1* (2.8x and 3.4x fold increase in KU812 and K562 cells) while *PIM1* and *CISH* gene expression were downregulated after IM treatment (Figure 3A). These results were also confirmed by Western blot analysis (Figure 3B). Importantly, we also found that expressions of *CAT* and *GLRX1* were reduced in primary leukemic cells from CML patients at diagnosis compared to mononuclear cells from healthy donors (Figure 3C). These data indicated that Bcr-Abl signaling inhibits expression of both enzymes in CML cells. We next evaluated the contribution of STAT5 in the regulation of catalase and Glrx1 protein expression and found that RNA interference-mediated knockdown of STAT5 in Bcr-Abl^+^ leukemia cells increased the expression of catalase and Glrx1 (2 to 3 fold) (Figure 3D and Supplementary Figure S3A). The dominant negative Δ5A mutant also induced catalase protein expression and, as expected, inhibited Pim-1 expression in KU812 cells (Supplementary Figure S3B)

### Oncogenic STAT5 signaling promotes ROS production and down-regulation of catalase and Glrx1 in hematopoietic cells

**Figure 4:**
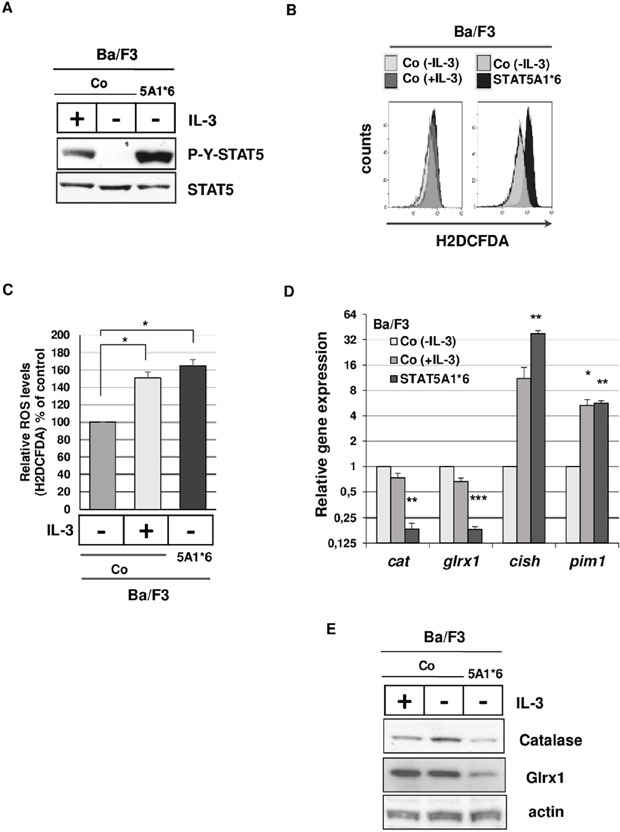
Tyrosine-phosphorylated STAT5 induces ROS production and inhibits catalase and Glrx1 expression in Ba/F3 cells **A**. Extracts prepared from Ba/F3 cells stimulated or not with IL-3 and from Ba/F3 cells stably expressing STAT5A1*6 were analyzed by Immunoblotting with indicated antibodies. Results are the mean of 3 independent experiments. **B**. Representative flow cytometry histogram of Ba/F3 cells stimulated (+IL-3) or not (−IL-3) with IL-3 and Ba/F3 cells expressing STAT5A1*6 mutant. Cells were incubated with the ROS sensitive fluorescent probe H2DCFDA (5 μM) and intracellular ROS levels were determined by flow cytometry. **C**. Statistical analysis showing relative ROS levels (% of control) detected in Ba/F3 cells stimulated or not with IL-3 and Ba/F3 cells expressing STAT5A1*6 (n=7, data are mean ± SEM. *p<0.05). **D**. qRT-PCR analysis of *cat*, *glrx1*, *cish* and *pim1* transcriptsin Ba/F3 cells transformed by constitutively active STAT5A1*6 mutant and control Ba/F3 cells grown in presence or absence of IL-3 (4h starvation). Results are presented as fold changes in gene expression in Ba/F35A1*6 and control Ba/F3 cells (+IL-3) normalized to internal control genes (*gapdh*) and relative to control Ba/F3 cells (−IL-3) normalized to 1 (n=3 in triplicates, data are mean +/− SEM. ***p<0.001; **p<0.01; *p<0.05). **E**. Expression of catalase and Glrx1 in Ba/F35A1*6 and control Ba/F3 cells grown in the presence or not of IL-3 (4hr starvation) as determined by Western blot analysis. Actin served as a loading control (n=3).

To confirm that persistent STAT5 activity is required for this inhibitory effect, we used Ba/F3 cells transformed by a constitutively active STAT5A1*6 mutant (Ba/F35A1*6). We first measured ROS levels in Ba/F35A1*6 and control Ba/F3 cells. Constitutive tyrosine phosphorylation of STAT5 and higher ROS levels were evidenced in Ba/F35A1*6 cells compared to IL-3-deprived control cells (Figure 4A-4C). Tyrosine phosphorylation of STAT5 and ROS level were also enhanced by IL-3 in control cells. The antioxidant gene expression profile was then determined in Ba/F35A1*6 cells by qRT-PCR assays using murine primers (Supplementary Table S2). Results showed that only *cat* and *glrx1* expressions were affected in these transformed cells (Supplementary Figure S4). Levels of *cat* and *glrx1* mRNAs and proteins were found to be decreased while expression of *pim1* and *cish* control genes were strongly induced in Ba/F35A1*6 cells (Figure 4D, 4E). Collectively, these data supported our findings that oncogenic activation of STAT5 triggers ROS production through mechanisms involving inhibition of catalase and Glrx1 expression.

### Catalase and Glrx1 reduce ROS production in Ph^+^ leukemia cells

**Figure 5:**
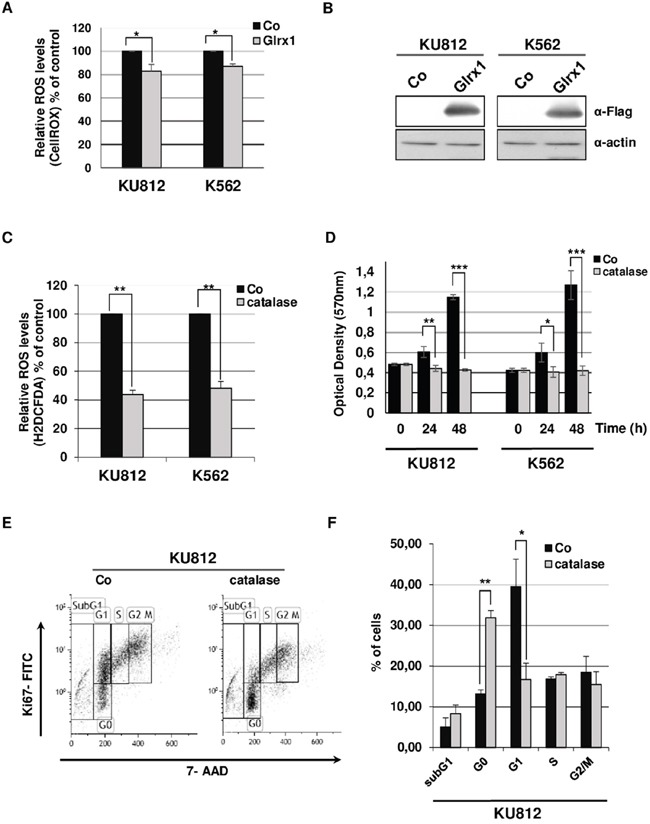
Effects of catalase and Glrx1 on ROS production and proliferation of Bcr-Abl+ leukemia cells **A**. KU812 and K562 cells were transfected with Glrx1/GFP (Glrx1) or empty/GFP (Co) expression vectors. At 2 days post transfection, cells were stained with CellROX to detect ROS levels in GFP^+^ cells (n=3, data are mean ± SEM. * p <0.05). **B**. Extracts from transfected cells were subjected to Western blot analysis to evaluate expression of the Flag-Glrx1 protein with an anti-Flag antibody. Actin served as loading control. **C**. KU812 and K562 cells were cultured for 48h in the presence or not of catalase (0.5 mg/ml). Cells were then stained with H2DCFDA to measure intracellular ROS levels (n=3, data are mean ± SEM. ** p <0.01). **D**. Growth kinetics of KU812 and K562 cells cultured in absence (Co) or presence of catalase (0.5 mg/ml) were determined by MTT assays (n=3 in triplicates, data are mean ± SEM *p <0.05; **p <0.01;***pp<0.001). **E**. KU812 cells exposed or not (Co) to catalase were stained with 7-amino-actinomycin D (7-AAD) and an Alexa Fluor H488-conjugated anti-Ki67 antibody. Cell cycle phase distributions were then estimated by flow cytometry. One representative experiment is shown. **F**. The histogram presents the percentage of KU812 cells exposed or not (Co) to catalase in sub-G1 (apoptotic fraction) and in each phase of the cell cycle. (n=3, data are mean ± SEM. ** p <0.01;* p<0.05)

Glrx1 is a glutathione-dependent enzyme that maintains and regulates the cellular redox state and redox-dependent signaling pathways while catalase converts the ROS hydrogen peroxide (H_2_0_2_) to water and oxygen [30]. We therefore analyzed the requirement of these two proteins in the regulation of ROS levels in Bcr-Abl^+^ cells. A bicistronic vector carrying a Flag-Glrx1 and the GFP was introduced in KU812 and K562 cells and ROS levels were determined in GFP^+^ cell populations after staining with CellROX. Forced expression of Glrx1 induced a moderate but significant reduction of ROS levels in both cell types (17% and 15% decrease in KU812 and K562 cells respectively) (Figure 5A, 5B). The presence of catalase is mainly detected in the cytoplasm and peroxisomes but reports also indicated that catalase is a membrane-bound extracellular enzyme [31]. We added catalase to the culture medium of K562 and KU812 cells and next measured intracellular ROS levels to find that catalase strongly reduced ROS levels in both leukemic cell lines (56% and 51% decrease in KU812 and K562 cells respectively) (Figure 5C).

### Catalase induces growth arrest and quiescence of Ph^+^ leukemia cells

The strong reduction of ROS level induced by exogenous catalase in Bcr-Abl-expressing cells prompted us to analyze the impact of this enzyme on cell growth. We observed that KU812 and K562 cells stopped dividing after catalase addition (Figure 5D). Cell viability and apoptosis were not affected but changes in cell cycle phase distributions were observed (Supplementary Figure S5A-S5C). Catalase increased the number of cells in the G0 phase with a concomitant decrease in the number of G1 cells, providing evidence that KU812 and K562 cells enter quiescence into the G0/G1 phase (Figure 5E, 5F and Supplementary Figure S5D). We verified the specificity of catalase activity in our experiments using 3-amino-1,2,4-triazole (3-AT), a catalase inhibitor. We showed that addition of 3-AT in the culture medium reduced the effect of catalase on ROS levels and leukemic cell quiescence (Supplementary Figure S6).

### STAT5-mediated oxidative stress is reduced in quiescent Ph^+^ leukemia cells

**Figure 6:**
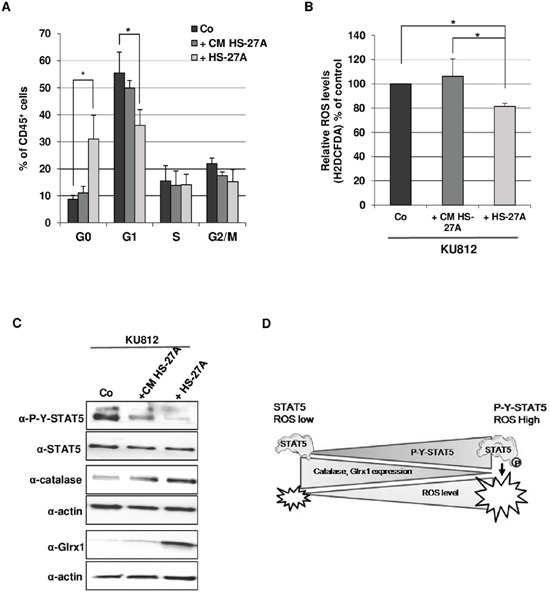
Contact with stromal cells promotes quiescence and reduction of STAT5-mediated oxidative stress in Bcr-Abl+ leukemia cells **A**. KU812 (10^5^ cells/ml) cells were cultured alone in medium (Co) or in HS-27A conditioned medium (+CM HS-27A) or on HS-27A cell monolayers for 72 h (+HS-27A). Cells were stained with 7-AAD and an Alexa Fluor H488-conjugated anti-Ki67 antibody. Concomitant staining with an APC-conjugated anti-CD45 antibody was performed to distinguish leukemic and stromal cells. Cell cycle phase distributions were estimated by flow cytometry. The histogram presents the percentage of leukemic cells (CD45^+^ cells) in the sub-G1 (apoptotic fraction) and cell cycle phases (n=3, data are mean ± SEM. *p<0.05). **B**. Cells were stained with the ROS sensitive dye H2DCFDA (5 μM) and an APC-conjugated anti CD45 antibody. ROS levels were then determined in leukemic cells (CD45^+^ cells) by FACS analysis (n=3 data are mean ± SEM. * p <0.05). **C**. KU812 cells cultured alone without (Co) or with HS-27A conditioned medium (+CM HS-27A) or on HS-27A cell monolayers (+HS-27A) were isolated using an immunomagnetic CD45 selection kit. KU812 cell extracts were then prepared and subjected to Western blot analysis with indicated antibodies. (n=3). **D**. Hypothetical model for the role of STAT5 as an inducer of ROS production in CML cells. The pro-oxidant activity of STAT5 is regulated by tyrosine phosphorylation. Constitutive activation of STAT5 (P-Y-STAT5) promotes oxidative stress by repressing expression of catalase and Glrx1. Dephosphorylation of STAT5 might allow the re-expression of catalase and Glrx1 and the decrease of ROS levels in leukemic cells.

It is well established that the maintenance of quiescent leukemic and normal hematopoietic stem cells is dependent on the bone marrow microenvironment and low ROS levels, suggesting that bone marrow stromal cells might deliver signals that could regulate intracellular ROS production and signaling pathways in leukemic cells [32–35]. We then designed experiments in which KU812 cells were cultured over human stromal cell monolayers using HS-27A cells, a bone marrow-derived stromal cell line [36]. KU812 cells were co-cultured with HS-27A cells during three days or grown alone in medium or conditioned medium from HS-27A cells as controls. Cell cycle analysis was performed on leukemic cells and showed that the contact with HS-27A cells increases the number of quiescent/G0 leukemic cells (Figure 6A). ROS level was also measured and found to be reduced in KU812 cells co-cultured with stromal cells (Figure 6B). No effect was observed when leukemic cells were grown in conditioned media from HS-27A indicating that contact with stromal cells is required for both induction of quiescence and downregulation of ROS level. Finally, we analyzed whether HS-27A cells could directly affect STAT5 phosphorylation, catalase and Glrx1 expression in leukemic cells. Western blot analysis showed a decrease in STAT5 phosphorylation which was accompanied by an upregulation of catalase and Glrx1 expression (Figure 6C). Interestingly, conditioned media from HS-27A was also able to slightly reduce STAT5 phosphorylation and to weakly increase catalase and Glrx1 expression. These data indicated that tyrosine phosphorylation is probably a key determinant of STAT5-mediated repression of catalase and Glrx1 and oxidative stress in Bcr-Abl-expressing cells. They also suggest that catalase expression by reducing ROS level might favour quiescence of leukemic cells in contact with stromal cells.

## DISCUSSION

Oxidative stress has been found in many cancers, including hematologic malignancies and elevated ROS levels have been detected in hematopoietic cells transformed by JAK2^V617F^, FLT3-ITD and Bcr-Abl oncogenes [21–23]. STAT5 was shown to regulate ROS production in leukemic cells with opposite effects and through distinct mechanisms that remain largely unclear [21, 25–28]. Our present work provides compelling evidence that activation of STAT5 induced by Bcr-Abl promotes oxidative stress in CML cells by repressing catalase and Glrx1 expression. Moreover, we found that expression of a constitutively active and oncogenic STAT5A mutant in hematopoietic cells is sufficient to observe this repression. Inhibition of both enzymes occurs at mRNA and protein levels indicating that STAT5 might directly or indirectly regulate expression of *CAT* and *GLRX1* genes. The inhibitory effect of the dominant negative STAT5AΔ749 mutant on the regulation of ROS level and repression of catalase would suggest that transcriptional activity of STAT5 is necessary for the regulation of both enzymes [37]. The JAK2^V617F^ oncogene was recently shown to repress catalase expression via a PI-3kinase/Akt-dependent pathway in murine hematopoietic cells and human CD34^+^ cells [22]. It is then conceivable that STAT5 and PI3-Kinase/Akt signalling act in concert to repress catalase expression in leukemic cells transformed by Bcr-Abl. Previous works illustrating a STAT5/PI3-kinase interaction in myeloid leukemias are in line with this putative regulatory mechanism [38]. Our data demonstrated that Glrx1 and catalase reduced ROS levels in Bcr-Abl-expressing cells. Glrx1 belongs to the family of glutaredoxins which utilize the reducing power of glutathione to catalyse disulfide reductions and protein deglutathionylation [39]. Glrx1 is the most abundant isoform and regulates the redox status of many proteins involved in signal transduction. However, the role of Glrx1 in the regulation of ROS production and protein glutathionylation in TKOs signalling is currently unknown. Reports indicated that oxidative stress promotes the S-glutathionylation of signalling proteins such as NF-KB and STAT3 [40, 41]. The downregulation of Glrx1 expression observed in Bcr-Abl^+^ cells might then increase the redox sensitivity of these proteins. In contrast to the moderate effect of Glrx1 on ROS levels, catalase was found to be as efficient as Imatinib to reduce intracellular ROS production in Bcr-Abl^+^ cells. Previous reports have shown that Bcr-Abl induced ROS accumulation in leukemic cells via activation of NADPH oxidases and a Rac2/MRC-cIII (Mitochondrial Respiratory Chain complex III) pathway [15, 42]. These data suggest that catalase is probably an important detoxifying enzyme able to eliminate ROS generated by both types of sources in Bcr-Abl-expressing cells. They also strengthen our finding that downregulation of catalase expression is probably essential in ROS accumulation induced by constitutively active STAT5 proteins in leukemic cells and most importantly in primary patient-derived CML cells. Interestingly, we found that catalase not only reduced ROS levels but also induced growth arrest, driving Bcr-Abl-expressing cells into a G0/quiescent state. These data indicate that ROS production directly affects Bcr-Abl-induced signaling pathways that are crucial for cell cycle progression. In support of this, overexpression of catalase has been shown to regulate cell cycle in vascular muscle smooth cells [43]. The contribution of catalase in the regulation of quiescence is also underscored by data obtained from the co-culture experiments aiming to mimic the bone marrow microenvironment of leukemia cells. We show that interaction with stromal cells promotes a decrease in STAT5 phosphorylation followed by an increase in catalase and Glrx1 expression and a reduction of ROS level in quiescent Bcr-Abl^+^ cells. These data also support our findings that repression or activation of catalase and Glrx1 expression and consequently ROS production are regulated by the fine tuning of STAT5 activity through phosphorylation/dephosphorylation-dependent mechanisms. Interestingly, catalase and STAT5 were previously shown to promote self-renewal and/or quiescence of normal hematopoietic stem cells as well as leukemic stem cells [44–47]. Our results suggest that stromal-derived signals induce quiescence of Bcr-Abl^+^ leukemia cells in part, through a STAT5/catalase dependent pathway. In sharp contrast to these data and those published by others [25], reports indicated that STAT5A mediates a protective role against oxidative stress in CML stem cells/progenitors [27]. We also reported a similar antioxidant activity of STAT5A in pre-B leukemic cells [28]. However, we showed that this effect was only observed in cells expressing a non-tyrosine(Y694)-phosphorylated form of STAT5A. The dephosphorylation of STAT5 as it is observed in Bcr-Abl-expressing cells when co-cultured with stromal cells might promote the protective role of the non-tyrosine-phosphorylated protein against an overproduction of ROS.

As summarized in Figure 6D, our work provides compelling evidence that sustained tyrosine phosphorylation of STAT5 induced by Bcr-Abl oncogene promotes oxidative stress in CML cells by repressing expression of two detoxifying enzymes, catalase and Glrx1. The dephosphorylation of STAT5 relieves this repression and consequently leads to the reduction of ROS production. Dephosphorylation might also unveil the protective role of STAT5 against oxidative stress through mechanisms that remain to be investigated.

## MATERIALS AND METHODS

### Cell culture and reagents

Cell lines were obtained from the American Type Culture Collection (ATCC) and maintained according to the supplier's recommendations. Ba/F3 cell lines expressing STAT5 mutants were previously reported [48]. Imatinib mesylate (IM) and catalase were purchased from Novartis (Basel, Switzerland) and Sigma-Aldrich (Lyon, France) respectively.

### CML patient samples

Leukemic cells from peripheral blood samples (35 adult patients) collected at diagnosis (before treatment) and peripheral mononuclear cells from 10 healthy volunteers were used to analyze *CAT* and *GLRX1* gene expression. All samples were obtained after informed consent and approval by the ethics committee of the University Hospital of Tours.

### Co-cultures and magnetic cell separation

HS-27A stromal cells were grown up to confluence in 150 cm^2^ flask. Medium was changed one day before starting the co-culture. 2.5 x10^6^ KU812 cells were next added on stromal cell monolayers for 72 hours. To discriminate between stromal cells and leukemic cells in apoptosis, cell cycle and ROS measurement analysis by flow cytometry, non-adherent and adherent leukemic cells were first labelled with an APC-conjugated anti-CD45 antibody (Becton-Dickinson). Leukemic cells were also purified by immunomagnetic selection using the EasySep PE positive selection kit (StemCell Technologies). Isolated CD45^+^ cells were then used to determine protein expression according to Western blot procedure.

### Plasmids and transfections

The coding regions of the dominant negative Stat5aΔ749 cDNA was amplified by PCR and cloned and subcloned in the pMACS4-IRESII vector (Miltenyi Biotec, Paris, France). Glutaredoxin-1 cDNA was amplified by PCR and cloned at NotI/SalI of the pIRES-hrGFP1a vector.

Short hairpins against human Stat5a/5b genes (shST5: GGAGAACCTCGTGTTCCTG) was subcloned at the BbsI site of psiRNA-h7SK-GFPzeo vector (Invivogen, Toulouse, France). For control, a shRNA targeting firefly luciferase was used (psiRNA-h7SK-GFPzeo-luc).

For transient transfection assays, cells were electroporated (270V, 960μF) with the different constructs (50 μg). Transfected cells were expanded for 24-48 hours in medium before analysis.

### Analysis of ROS levels

Intracellular reactive oxygen species (ROS) production was measured using CellROX Deep Red Reagent (life technologies, Saint Aubin, France) or 5-(and-6)-chloromethyl-2′, 7′-dichlorodihydrofluorescein diacetate, acetyl ester (CM-H2DCFDA) (Invitrogen, Carlsbad, United States). Cells (0.5×10^6^) were washed with PBS (phosphate-buffered saline) and stained with 5μM CellROX for 30 min or 5μM CM-H2DCFDA for 10 min at 37°C. Cells were then analyzed by flow cytometry on a *Beckman Coulter* Gallios *or a Becton Dickinson Accuri™ C6 flow cytometer*.

### Cell cycle and apoptosis analysis

Cells were stained for 30 min at room temperature with 7-amino-actinomycin D (7-AAD) (Sigma-Aldrich, Lyon, France) and anti-Ki67-Alexa Fluor 488 monoclonal antibody (Becton-Dickinson, Franklin Lakes, United States) as previously described [49]. For apoptosis studies, cells were stained (0,25 × 10^6^ cells) in buffer containing FITC-AnnexinV and 7-amino-actinomycin D (7-AAD) (Beckmann Coulter, Fullerton, United States) for 15 min at 4°C and then analyzed by FACS.

### qRT-PCR analysis

RNA samples were reverse-transcribed using SuperScript®VILO cDNA Synthesis kit (Invitrogen, Carlsbad, United States) as recommended by the supplier. The resulting cDNAs were used for quantitative real-time PCR (qRT-PCR). PCR primers (Supplementary Tables S1 and S2) were designed with the ProbeFinder software (Roche Applied Sciences, Basel, Switzerland) and used to amplify the RT-generated cDNAs. qRT-PCR analyses were performed on the Light Cycler 480 thermocycler II (Roche) Both *GAPDH* (glyceraldehyde-3-phosphate dehydrogenase) and *ACTB* (actin beta) were used as reference genes for normalization of qRT-PCR experiments. Each reaction condition was performed in triplicate. Relative gene expression was analyzed using the 2^−ΔΔCt^ method [50].

### Western blotting and antibodies

NP40 cell lysates (1% NP40, 10% glycerol, 0.05 M Tris pH 7.5, 0.15 M NaCl, 1 mM PMSF, protease and phosphatase Inhibitor cocktails) (Roche and Thermo Scientific, respectively) were resuspended in Laemmli's 2x buffer, separated on SDS/PAGE and blotted onto nitrocellulose membrane (GE Healthcare, Little Chalfont, United Kingdom). Blots were incubated with the following antibodies (Abs): P-Y^694/699^-STAT5, catalase (Cell Signaling Technology, Danvers, United States), STAT5 (BD Transduction Laboratories, Franklin Lakes, United States), actin (Santa Cruz, Dallas, United States), Flag M2 (Stratagene, Santa Clara, United States), Glutaredoxin1 (Glrx1) (R&D, Minneapolis, United States). Membranes were developed with the ECL chemiluminescence detection system (GE Healthcare, Little Chalfont, United Kingdom) using specific peroxidase (HRP) conjugated to rabbit or mouse IgG antibodies (Cell signaling Technology, Danvers, United States) or goat IgG (Santa Cruz, Dallas, United States).

### MTT assays

Cell viability and proliferation were studied using an MTT cell proliferation assay. Briefly, 0.5×10^5^ cells were incubated in 100μl of X-Vivo red phenol free medium (Lonza, Basel, Switzerland) in 96 well plates. Cells were treated with 10 μl of MTT working solution (5g/l of Methylthiazolyldiphenyl-tetrazolium bromide) during 4 hours. Cells were then lysed overnight at 37°C with 100 μl of SDS 10%, HCl 0.003%. Optical density (OD) at 570nm was measured using a spectrophotometer (Dynex, Chantilly, United States).

### Statistical analysis

Data are presented as mean ± SEM (n= number of individual measurements). The Student's t-test was used to compare two means and to determine significant differences. Differences between values were considered significant (*) when p< 0.05; very significant (**) when p< 0.01 and highly significant (***) when p< 0.001. All calculations were performed using the GraphPad Prism 6 software.

## SUPPLEMENTARY FIGURES AND TABLES




