# Supplementary material for: Oncogenic STAT5 signaling promotes oxidative stress in chronic myeloid leukemia cells by repressing antioxidant defenses
Source: Oncotarget. 2016 Aug 22;8(26):41876–89. doi: 10.18632/oncotarget.11480 (PMC5522035; doi:10.18632/oncotarget.11480)
Supplement: Supplementary file 1 [file oncotarget-08-41876-s001.pdf]

Oncogenic STAT5 signaling promotes oxidative stress in chronic myeloid leukemia cells by repressing antioxidant defenses

SUPPLEMENTARY FIGURES AND TABLES

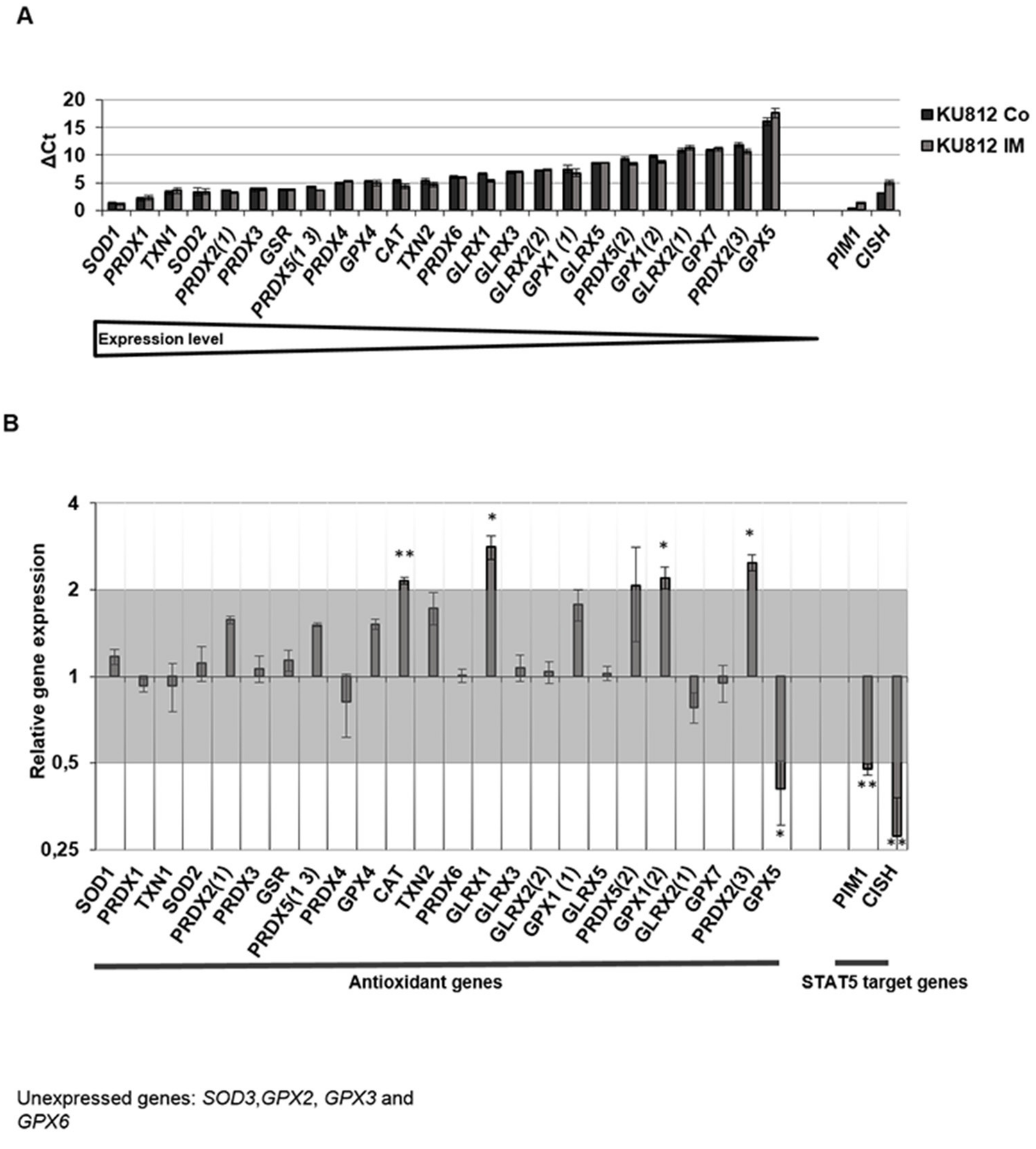

Supplementary Figure S1: A. Antioxidant gene expression profile in KU812 cells treated or not with IM (1µM) for 15 h. Genes were ranked according to their expression levels in untreated cells (ΔCt values). Gene expression data in KU812 cells treated or not with IM were normalized to internal control genes (*GAPDH* and *ACTB*). Expression analysis of two STAT5 target genes, *PIM1* and *CISH*, were also included in the qRT-PCR studiesas positive controls (n=3 in triplicates, data are mean + / - SEM). **B.** Regulation of antioxidant gene expression by IM in KU812 cells. Results are presented as fold changes in gene expression in IM-treated cells ($2^{-\Delta\Delta Ct}$values) relative to untreated cells (normalized to 1). Mean values ranging from 0,5 to 2 (grey area) were not considered as significant changes. Genes with low or very low levels of expression (*GPX1*, *PRDX2*, *GPX5*) were also not considered as significant changes. IM-dependent downregulation of positive control gene expressions*: PIM1* and *CISH* is also shown (n=3 in triplicates, data are mean + / - SEM, **p<0,01; *p<0,05).

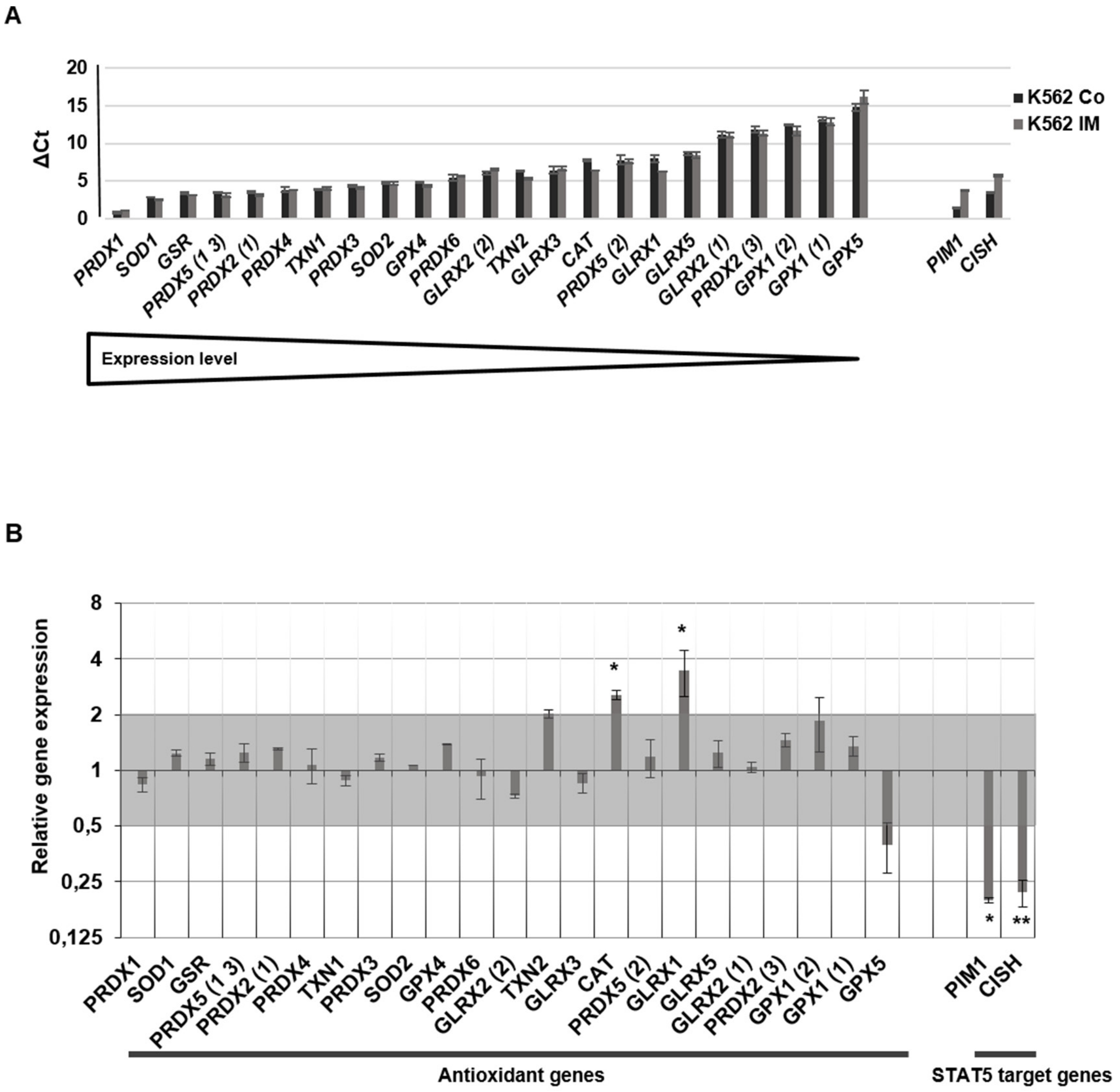

Unexpressed genes: *SOD3*,*GPX2*, *GPX3*, *GPX6* and *GPX7*

Supplementary Figure S2: A. Antioxidant gene expression profile in K562 cells treated or not with IM (1µM) for 15 h. Genes were ranked according to their expression levels in untreated cells (ΔCt values). Gene expression data in K562 cells treated or not with IM were normalized to internal control genes (*GAPDH* and *ACTB*). Expression analysis of two STAT5 target genes, *PIM1* and *CISH,* were also included in these qRT-PCR studies as positive controls (n=3 in triplicates, data are mean + / - SEM). **B.** Regulation of antioxidant gene expression by IM in K562 cells. Results are presented as fold changes in gene expression in IM-treated cells ($2^{-\Delta\Delta Ct}$values) relative to untreated cells (normalized to 1). Mean values ranging from 0,5 to 2 (grey area) were not considered as significant changes. Genes with low or very low levels of expression (*GPX5*) were also not considered as significant changes. IM-dependent downregulation of positive control gene expressions*: PIM1* and *CISH* is also shown (n=3 in triplicates, data are mean + / - SEM, **p<0,01; *p<0,05).

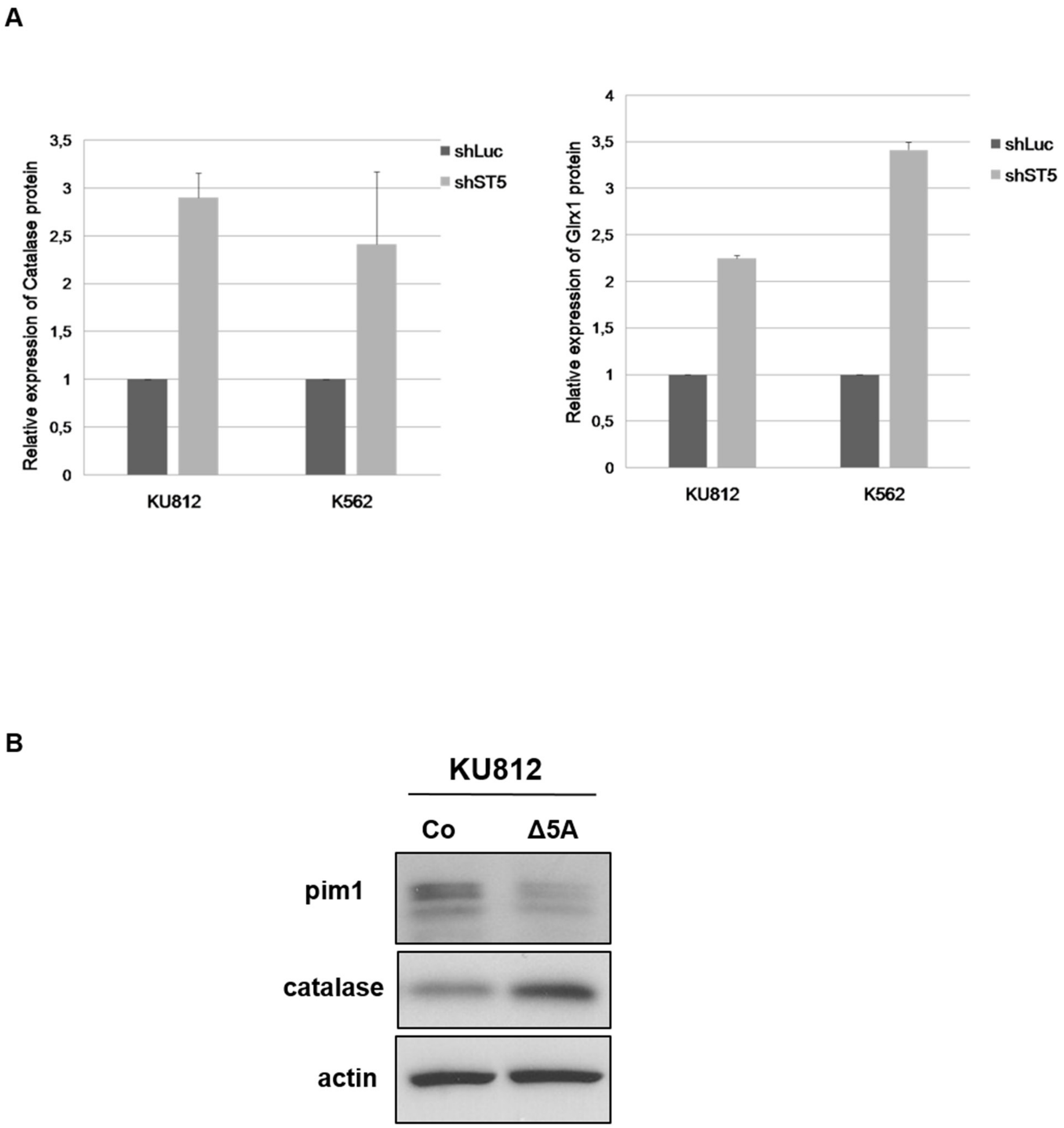

Supplementary Figure S3: A. The relative expression of catalase (left panel; ratio catalase/actin; n=3) and Glrx1 (right panel; ratio Glrx1/actin; n=3) in KU812 and K562 cells transfected with shST5/GFP or shLuc/GFP vectors were evaluated by band intensity quantification. **B.** Extracts from KU812 cells transfected with the dominant negative STAT5A (Δ5A) construct or an empty vector (Co) were prepared and analyzed by Western blot to determine the expression level of pim1 and catalase proteins. Actin served as a loading control.

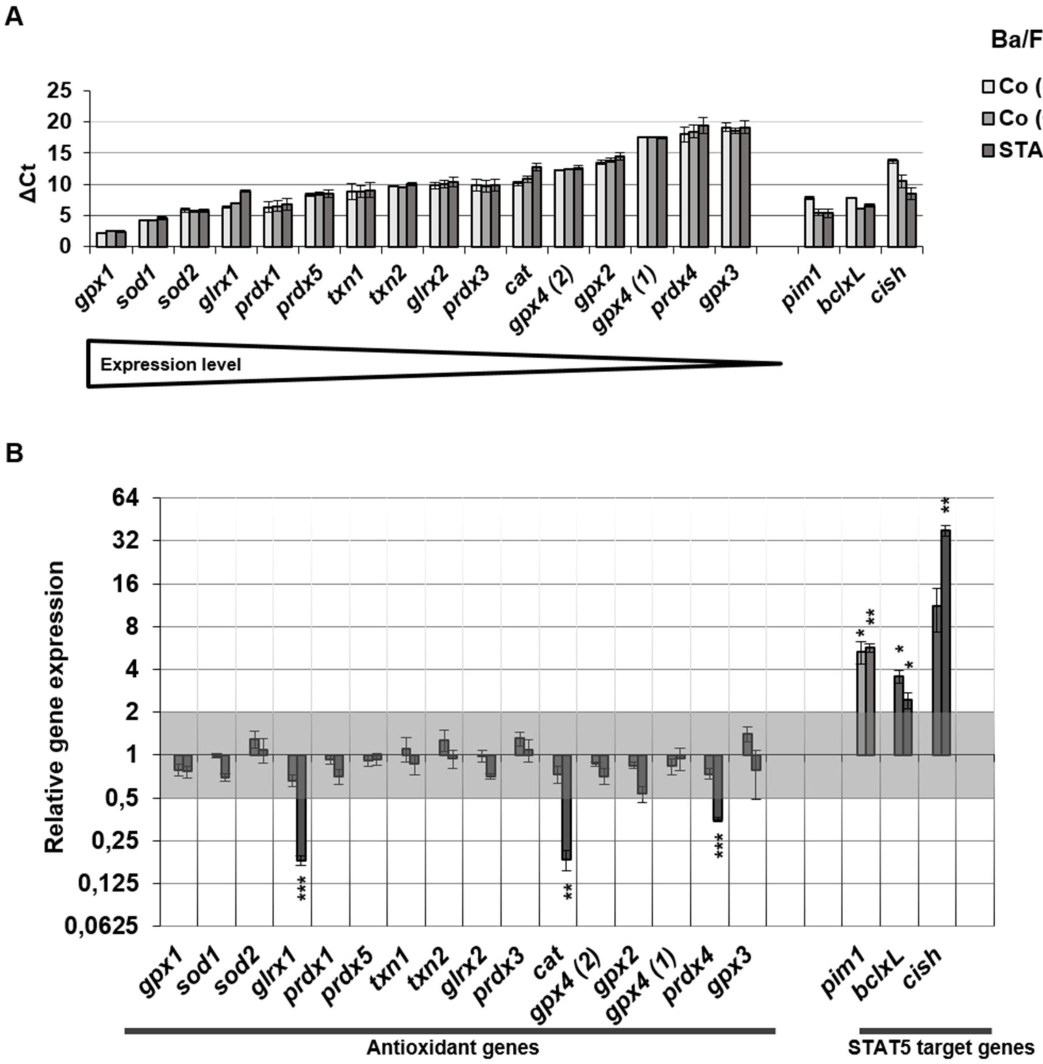

Supplementary Figure S4: A. Antioxidant gene expression profile in Ba/F3 cells grown in absence or presence of IL-3and Ba/F3 cells transformed by constitutively active STAT5A1*6 mutant. Genes were ranked according to their expression levels in IL-3-starved cells (ΔCt values). Gene expression data for each cell lines or conditions were normalized to internal control gene (*gapdh)*. Expression analysis of threeSTAT5 target genes, *pim1, bcl-x* and *cish,* were also included in these qRT-PCR studies as positive controls (n=3, data are mean + / - SEM). **B.** Regulation of antioxidant gene expression by constitutively active STAT5 mutants in Ba/F3 cells. Results are presented as fold changes in gene expression in IL-3 stimulated cells (Co, +IL-3) and cells transformed by STAT5A1*6 mutants ($2^{-\Delta\Delta Ct}$values) relative to IL-3-starved cells (Co, -IL-3) (normalized to 1). Mean values ranging from 0,5 to 2 (grey area) were not considered as significant changes. Genes with low or very low levels of expression (*prdx4*) were also not considered as significant changes. Induction of *pim1, bcl-x* and *cish* gene expression by IL-3 or by active STAT5A1*6is also shown (n=3 in triplicates, data are mean + / - SEM, **p<0,01; *p<0,05).

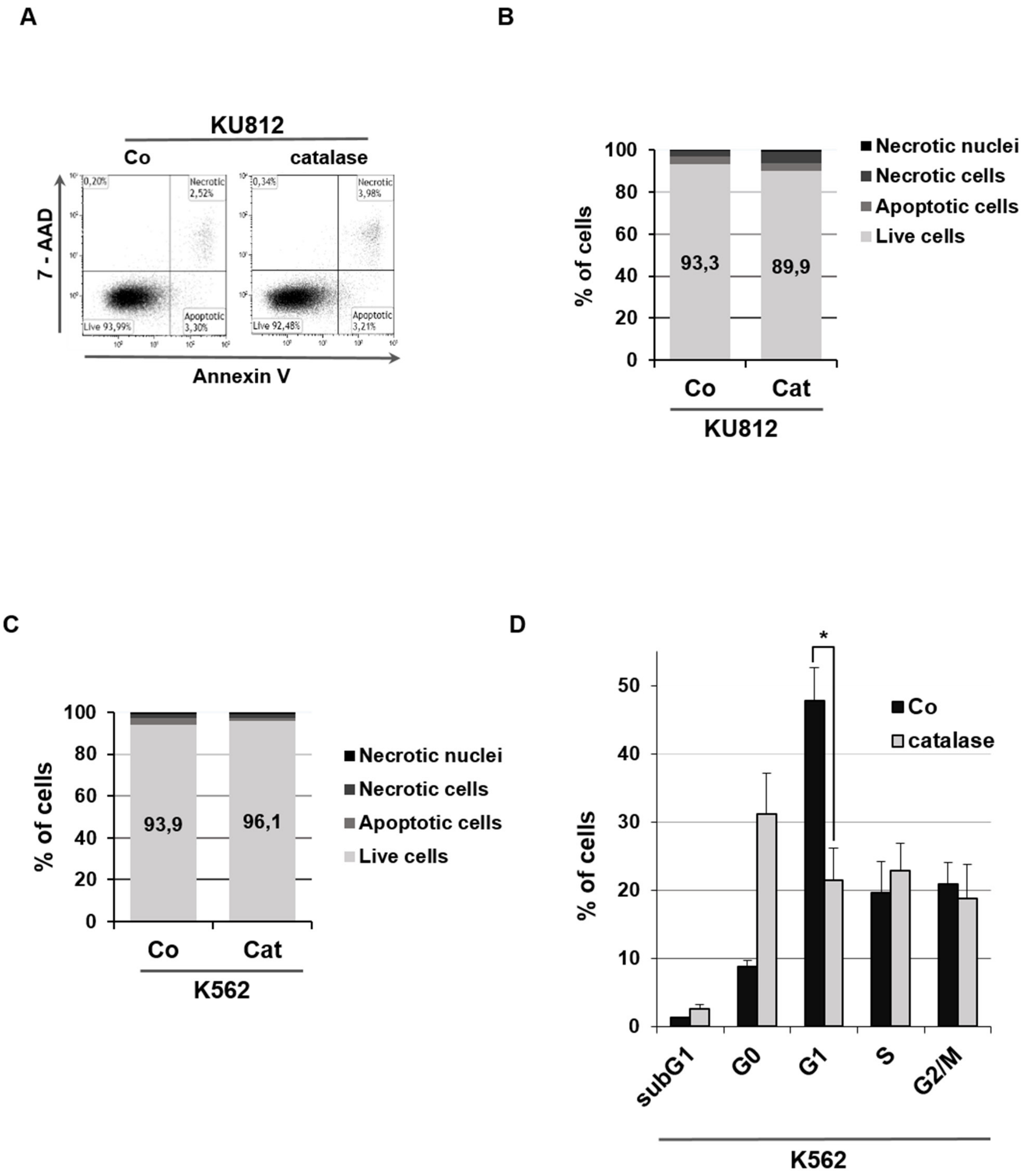

Supplementary Figure S5: A. Representative flow cytometry histogram of KU812 cells cultured for 48 h in the presence or not (Co) of catalase (0,5mg/ml). Cells were stained with FITC-Annexin V and 7-AAD and the percentages of apoptotic and necrotic cellswerenext evaluated by FACS analysis. **B.** and **C.** Statistical analysis showing the percentages of apoptotic and necrotic KU812 cells (B) or K562 cells (C) cultured (Cat) or not (Co) with catalase (n=3, no significant difference). **D.** K562 cells cultured with or without (Co) catalase during 48hr were stained with 7-AAD and anAlexa Fluor H488-conjugated anti-Ki67 antibody. Cell cycle phase distribution was then estimated by flow cytometry. The histogram presents the percentage of K562 cells in sub-G1 (apoptotic fraction) and in each phase of the cell cycle (n=3, data are mean ± SEM, * $p<0,05$).

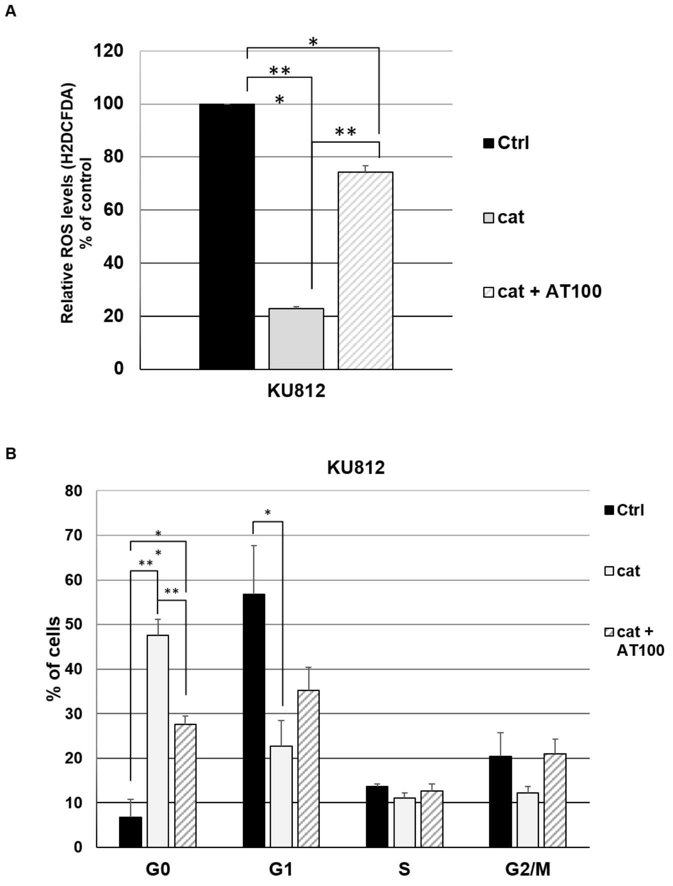

Supplementary Figure S6: A. KU812 cells were cultured for 24 h in the presence or not (Ctrl) of catalase (0.5 mg/ml) with (AT100=100 µM) or without 3-AT. Cells were then stained with H2DCFDA to measure intracellular ROS levels (n=3, data are mean ± SEM. * p <0.05; ** p <0.01; ***pp<0.001). **B.** KU812 cells exposed or not (Ctrl) to catalase with (AT100=100 µM) or without 3-AT were stained with 7-amino-actinomycin D (7-AAD) and an Alexa Fluor H488-conjugated anti-Ki67 antibody. Cell cycle phase distributions were then estimated by flow cytometry.

Supplementary Table S1: Human primers and UPL probes used in qRT-PCR studies

See Supplementary File S1

Supplementary Table S2: Murine Primers and UPL probes used in qRT-PCR studies

Gene Symbol	Gene name	Primer Sequence		UPL probes
		forward	Reverse	
bclxl	bcl2-like 1	tgaccacctagagccttgga	tgttcccgtagagatccacaa	#2
cat	catalase	gcgaccagatgaagcagtg	gtggtcaggacatcaggtctc	#68
cish	cytokine inducible SH2-containing protein	gacatggtcctttgcgtaca	atgccccagtgggtaagg	#1
gapdh	glyceraldehyde-3-phosphate dehydrogenase	tgtccgtcgtggatctgac	cctgcttcaccaccttcttg	#80
glrx1	glutaredoxin 1	tggagctctgcagttataaaagg	gccatcagcatggttagaca	#13
glrx2	glutaredoxin 2	ctcctgtgaaccagatccaag	cttcttggccatggaacagt	#15
gpx1	glutathione peroxidase 1	tttcccgtgcaatcagttc	tcggacgtacttgagggaat	#2
gpx2	glutathione peroxidase 2	gttctcggcttcccttgc	tcaggatctcctcgttctgac	#2
gpx3	glutathione peroxidase 3	ggcttcccttccaaccaa	cccacctggtcgaacatact	#92
gpx4 (1)	glutathione peroxidase 4, (variant 1)	cgagttcctgggcttgtg	ttatccaggcagaccatgtg	#102
gpx4 (2)	glutathione peroxidase 4, (variant 2)	ccgtctgagccgcttactta	ctgagaattcgtgcatggag	#71
gpx6	glutathione peroxidase 6	cttctgtggcctgacagcta	cgtgacgttgaatggcttc	#63
gpx7	glutathione peroxidase 7	caccctgccttcaagtaccta	tttccgtctgggtccacta	#12
pim1	proviral integration site 1	cagtctacacggactttgatgg	cgcagaccatgtcatagagc	#93
prdx1	peroxiredoxin 1	gtgagacctgtggctcgac	tgtccatctggcataacagc	#15
prdx3	peroxiredoxin 3	gtgcctcttgcgtgctct	acttgcatgacgagcaacc	#93
prdx4	peroxiredoxin 4	gacgagacactgcgtttgg	gcagacttctccatgcttgtc	#97
prdx5	peroxiredoxin 5	ggcatttacacctggctgtt	gctcagacaggccaccac	#78
prdx6	peroxiredoxin 6	tttcaatagacagtgttgaggatca	cgtgggtgtttcaccattg	#1
sod1	superoxide dismutase 1, soluble	caggacctcattttaatcctcac	tgcccaggtctccaacat	#49
sod2	superoxide dismutase 2, mitochondrial	gacccattgcaaggaacaa	gtagtaagcgtgctcccacac	#3
sod3	superoxide dismutase 3, extracellular	ctcttgggagagcctgaca	gccagtagcaagccgtagaa	#102
txn1	thioredoxin 1	tgaagctgatcgagagcaag	agaagtccaccacgacaagc	#81
txn2	thioredoxin 2	atccctctgctcgcactg	ggagatgactgaggtcaggaac	#63
