# Supplementary material for: Oncogenic STAT5 signaling promotes oxidative stress in chronic myeloid leukemia cells by repressing antioxidant defenses
Source: Oncotarget. 2016 Aug 22;8(26):41876–89. doi: 10.18632/oncotarget.11480 (PMC5522035; doi:10.18632/oncotarget.11480)
Supplement: Supplementary file 2 [file oncotarget-08-41876-s002.docx]

| ***Gene Symbol*** | G**ene name** | Primer Sequence | | U**PL probes** |
| --- | --- | --- | --- | --- |
|  |  | **Forward** | **Reverse** |  |
| *ACTB* | β actin | attggcaatgagcggttc | cgtggatgccacaggact | #11 |
| *CAT* | Catalase | cgcagttcggttctccac | gggtcccgaactgtgtca | #67 |
| *CISH_var1&2* | cytokine inducible SH2-containing protein | agccaagaccttctcctacctt | tggcatcttctgcaggtgt | #20 |
| *GAPDH* | glyceraldehyde-3-phosphate dehydrogenase | agccacatcgctcagacac | gcccaatacgaccaaatcc | #60 |
| *GLRX (1&2)* | glutaredoxin 1 (thioltransferase) | ggcttctggaatttgtcgat | tgcatccgcctatacaatctt | #83 |
| *GLRX2 (1)* | glutaredoxin 2 (variant 1) | gtggcactcgctggaatc | cgtcgctaaattctccaaagat | #58 |
| *GLRX2 (2)* | glutaredoxin 2 (variant 2) | gctggtttggagcaggag | ccaaagatgatgatgtattgctct | #70 |
| *GLRX3* | glutaredoxin 3 | tcctcaagaaccacgctgt | tgagaagatatcaaaactgctaaactg | #31 |
| *GLRX5* | glutaredoxin 5 | gtgataactggggcgttgtt | actcaggcatgcacagca | #67 |
| *GPX1 (1)* | glutathione peroxidase 1, (variant 1) | caaccagtttgggcatcag | gttcacctcgcacttctcg | #77 |
| *GPX1 (2)* | glutathione peroxidase 1, (variant 2) | cccttgtttgtggttagaacg | gagagaagggcagctagaacc | #63 |
| *GPX2* | glutathione peroxidase 2 | gtccttggcttcccttgc | tgttcaggatctcctcattctg | #2 |
| *GPX3* | glutathione peroxidase 3 | cagagatccttcctaccctcaa | ccctttctcaaagagctgga | #39 |
| *GPX4 (1,2&3)* | glutathione peroxidase4,(variants1, 2 and 3) | tacggacccatggaggag | ccacacacttgtggagctagaa | #43 |
| *GPX5 (1&2)* | glutathione peroxidase 5,(variants 1 and 2) | tgctttgtgcaaacaagtcc | ggtgcctttctcgtctttgt | #2 |
| *GPX6* | glutathione peroxidase 6 | aggcttggcagctcagtatc | gccaacacaatgacaccaaa | #17 |
| *GPX7* | glutathione peroxidase 7 | ccatcctgccttcaagtacc | ttccatctggggctactagg | #12 |
| *GSR* | glutathione reductase | tgccagcttaggaataaccag | cctgcaccaacaatgacg | #11 |
| *PIM1* | pim-1 oncogene | tttcgagcatgacgaagaga | gggccaagcaccatctaat | #13 |
| *PRDX1 (1,2&3)* | peroxiredoxin 1, (variants 1, 2 and 3) | cactgacaaacatggggaagt | tttgctcttttggacatcagg | #20 |
| *PRDX2 (1)* | peroxiredoxin 2, (variant 1) | gccttccagtacacagacgag | gttgggcttaatcgtgtcact | #60 |
| *PRDX2 (3)* | peroxiredoxin 2, (variant 3) | gcaactcagatgcaactctatctact | tgaactggagtttccatcttcat | #75 |
| *PRDX3 (1&2)* | peroxiredoxin 3, (variant 1 and 2) | ctggacaccggattctccta | gggtgatctactgatttaccttctg | #38 |
| *PRDX4* | peroxiredoxin 4 | gcacctaagcaaagcgaaga | aaattctccatcgatcacagc | #48 |
| *PRDX5 (1&3)* | peroxiredoxin 5, (variants 1 and 3) | tcctggctgatcccactg | atgccatcctgtaccaccat | #12 |
| *PRDX5 (2)* | peroxiredoxin 5, (variant 2) | cacccctggatgttccaa | ggacaccagcgaatcatctagt | #12 |
| *PRDX6* | peroxiredoxin 6 | caatagacagtgttgaggaccatc | tttctgtgggctcttcacaa | #1 |
| *SOD1* | superoxide dismutase 1, soluble | gcatcatcaatttcgagcag | caggccttcagtcagtcctt | #60 |
| *SOD2* | superoxide dismutase 2, mitochondrial | tccactgcaaggaacaacag | taagcgtgctcccacacat | #3 |
| *SOD3* | superoxide dismutase 3, extracellular | ctctcttttcaggagagaaagctc | aacacagtagcgccagcat | #17 |
| *TXN* | thioredoxin | ttacagccgctcgtcaga | ggcttcctgaaaagcagtctt | #66 |
| *TXN2* | thioredoxin 2 | gagacaccagtggttgtgga | gcttggccaccatcttctc | #57 |

Table S1
